# Modeling of Non-Steroidal Anti-Inflammatory Drug Effect within Signaling Pathways and miRNA-Regulation Pathways

**DOI:** 10.1371/journal.pone.0072477

**Published:** 2013-08-14

**Authors:** Jian Li, Ulrich R. Mansmann

**Affiliations:** 1 Institute for Medical Informatics, Biometry and Epidemiology, Ludwig-Maximilians-University Munich, Munich, Germany; 2 German Cancer Consortium (DKTK), Heidelberg, Germany; 3 German Cancer Research Center (DKFZ), Heidelberg, Germany; University of Erlangen-Nuremberg, Germany

## Abstract

To date, it is widely recognized that Non-Steroidal Anti-Inflammatory Drugs (NSAIDs) can exert considerable anti-tumor effects regarding many types of cancers. The prolonged use of NSAIDs is highly associated with diverse side effects. Therefore, tailoring down the NSAID application onto individual patients has become a necessary and relevant step towards personalized medicine. This study conducts the systemsbiological approach to construct a molecular model (NSAID model) containing a cyclooxygenase (COX)-pathway and its related signaling pathways. Four cancer hallmarks are integrated into the model to reflect different developmental aspects of tumorigenesis. In addition, a Flux-Comparative-Analysis (FCA) based on Petri net is developed to transfer the dynamic properties (including drug responsiveness) of individual cellular system into the model. The gene expression profiles of different tumor-types with available drug-response information are applied to validate the predictive ability of the NSAID model. Moreover, two therapeutic developmental strategies, synthetic lethality and microRNA (miRNA) biomarker discovery, are investigated based on the COX-pathway. In conclusion, the result of this study demonstrates that the NSAID model involving gene expression, gene regulation, signal transduction, protein interaction and other cellular processes, is able to predict the individual cellular responses for different therapeutic interventions (such as NS-398 and COX-2 specific siRNA inhibition). This strongly indicates that this type of model is able to reflect the physiological, developmental and pathological processes of an individual. The approach of miRNA biomarker discovery is demonstrated for identifying miRNAs with oncogenic and tumor suppressive functions for individual cell lines of breast-, colon- and lung-tumor. The achieved results are in line with different independent studies that investigated miRNA biomarker related to diagnostics of cancer treatments, therefore it might shed light on the development of biomarker discovery at individual level. Particular results of this study might contribute to step further towards personalized medicine with the systemsbiological approach.

## Introduction

NSAIDs are a class of drugs with distinct chemical structures. However, they can invoke the common therapeutic effect: an anti-inflammatory and anti-neoplastic effect [1]. The key molecular mechanism for this type of anti-tumor drug is the inhibition of cyclooxygenase (COX) pathway, whose center components include cyclooxygenase-2 (COX-2), cytosolic glutathione transferases (GSTM2, 3), and prostaglandin E_2_ (PGE_2_). In this pathway, key steps are the enzymatic conversion from arachidonic acid to prostaglandin G_2_ (PGG_2_) catalyzed by COXs (COX-1 and -2) and subsequent conversion from PGG_2_ to prostaglandin H_2_ (PGH_2_) catalyzed by the same enzymes. Each downstream component (including PGE_2_, PGI_2_, PGD_2_, PGF_2_ and TXA_2_) derived from PGH_2_ has its unique biological functions to mediate inflammatory responses and to involve pathophysiological processes [2,3].

To date, it is widely recognized that NSAIDs can exert considerable anti-tumor effect regarding many types of cancers such as colon [4], lung [5], prostate [6], head-and-neck [7] and stomach [8]. It was estimated that the regular use of NSAIDs for a 10- or 15-year-period can reduce more than 40% of colon cancer occurrence [9]. Furthermore, it was estimated that in the USA alone, more than 20 billion aspirin (1st generation NSAID) tablets are purchased annually, and that more than 1% of the world population consumes at least one aspirin tablet daily [10]. Unfortunately, the frequent and prolonged use of NSAIDs has been associated with different adverse drug effects including gastritis, abdominal pain, peptic ulcer, gastrointestinal bleeding, nausea and others [11]. In order to minimize the drug’s side effects and produce high quality NSAIDs, it has been a chief interest to identify the NSAID related pathways as well as their physiological and pathological functions.

Until now, many studies have been conducted to reach the goal of understanding the molecular mechanism of NSAIDs, for instance, Dannenberg and Zakim [12] focused on the fact that the first generation of NSAIDs inhibit COX-1 and COX-2, which are the key enzymes responsible for the biosynthesis of prostaglandin from arachidonic acid and they discovered the diverse biological activities of prostagladins and the corresponding derived products; Fosslien [13] summarized that the activity of COX-2, which is undetected in most normal tissues, can be strongly induced by cytokines, growth factors, oncogenes, and tumor promoters. Those results indicate the carcinogenesis contribution of COX-2; subsequently, many studies discovered that PGE2 can invoke signaling cascades to perform crosstalk and synergistic effect with diverse signaling pathways such as epidermal growth factor receptor (EGFR)-signaling [14], nuclear receptor signaling [15], nuclear factor of kappa light polypeptide gene enhancer in B-cells (NfκB)-signaling [16], rat sarcoma (Ras)-mitogen activated protein kinase (MAPK) signaling [17,18], vascular endothelial growth factor (VEGFR)-signaling [19], janus kinase/signal transducer and activator of transcription (JAK-STAT)-signaling [20] and others.

While the details of NSAIDs molecular mechanisms have been elucidated, there is need to consider integrative systematic approaches for reconstruction and mathematical analyses of large-scale signaling networks related to NSAID. We want to investigate whether this type of modeling approach involving extracellular and intracellular signaling mechanisms might enable a system-level understanding of dynamic behavior of an individual cellular system. We also intend to investigate whether modeling of NSAIDs molecular mechanisms can help tailoring down the NSAID application to the individual level in order to predict the NSAID effect according to individuals.

Furthermore, biomarkers are defined as molecular, cellular or functional measurable parameters that can indicate a particular genetic, physiological or functional status of a cellular system [21]. To the present day, no effective *in silico* approach has been presented in order to discover high quality miRNA biomarkers. Therefore, we want to investigate whether the modeling of miRNA regulation networks could help us step further towards this goal.

For the first time, this study constructs a molecular model based on the literature information regarding COX-pathway and its related pathways. We named it the NSAID model containing 3874 components and 6398 biochemical reactions. Furthermore, we developed a Flux-Comparative Analysis (FCA) to incorporate individual genetic information and different kinetic parameter-values into the model for simulation. By applying this approach, we demonstrate the NSAID effect in silico and its further therapeutic potential applications with satisfactory results.

## Results

### Model Construction

**Figure 1:**
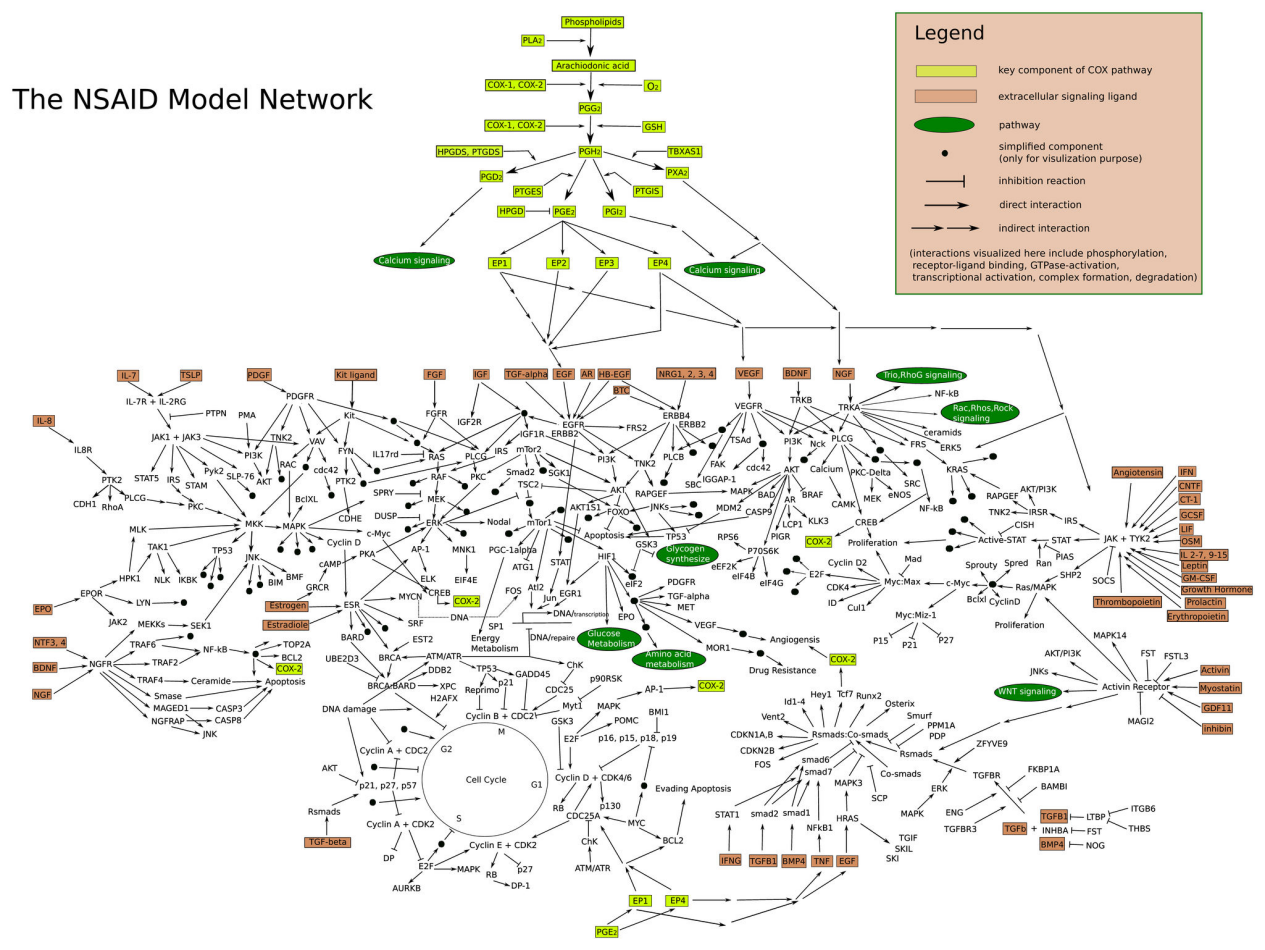
Simplified overview of the NSAID model.

The NSAID model contains three layers: a gene-layer, an RNA-layer and one other layer (includes protein, complex, metabolite). In the model, each gene takes part in a corresponding transcription reaction to generate its mRNA, which in turn produces its protein product via a corresponding translation. Transcription factors can be translocated into nucleoplasm to control their target genes by promoting/repressing transcription reactions (Supplementary Information S1). Therefore, all three layers in the model are inter-connected with each other. Table 1 summarizes the model components and reactions. The center part of this model is the COX-pathway, where it starts with the COX genes transcriptions. These lead to the COX protein products that bind to the available arachidonic acid in the model and catalyze its conversion into PGG2 under oxygen condition (Figure 1). PGG2 is then converted into the unstable intermediate PGH2 by the second enzymatic catalyzation of COXs. Afterwards, different prostaglandins (PGE_2_, PGI_2_, PGD_2_, PGF_2_ and TXA_2_) can be derived from PGH2 with the presence of corresponding tissue-specific prostaglandin synthases (PTGES, PTGIS, PTGDS, PTGFS and TBXAS1) [2]. The different prostaglandins can bind to corresponding receptors and the specific binding between prostaglandins and their receptors can invoke signaling cascades that are involved in diverse signaling pathways to exert cellular functions and signaling responses [3] (Figure 1). The model contains 20 signaling pathways and the Supplementary Information S2 lists literature and component information of each pathway in the model.

**Table 1:** The component/reaction summary of NSAID model.

**Component**	**No.**	**Reaction**	**No.**
gene	766	transcription	1145
mRNA	1530	translation	572
protein	1003	decay	1796
miRNA	18	complex-formation	363
compound	44	translocation	995
complex	486	phosphorylation	713
pseudo-object	21	dephosphorylation	212
siRNA	1	activation	240
		miRNA-binding	721
Sum:	3869	Sum:	6757

### Integration of Cancer Hallmarks

Hanahan and Weinberg [22,23] proposed different hallmarks of cancer and elucidated a framework for a biological organization principle of cancer developments, which in detail explains that the human cancer development is a multiple-step biological malignancy-process. Different independent studies show that the deregulation of COX-pathway can contribute to the tumorigenesis by at least four mechanisms: (1) sustained angiogenesis [24]; (2) tissue invasion and metastasis [25]; (3) proliferation [26]; (4) evading apoptosis [27], we have incorporated these four cancer hallmarks (sustained angiogenesis, invasiveness and metastasis, proliferation and apoptosis) into the NSAID model. The hallmark *proliferation* is defined in the model as the sum of putative proliferative biomarkers including upregulation of cell proliferation (URGCP), antigen identified by monoclonal antibody Ki-67 (MKI67), tripartite motif-containing protein 21 (TRIM21), DNA topoisomerase 2-alpha (TOP2A), forkhead box M1 (FOXM1), polo-like kinase 1 (PLK1) and others, which reflects the proliferative ability of the model and is directly embedded within the MAPK-, wingless-type MMTV integration site family (WNT)-, mechanistic target of Rapamycin (MTOR)-, and hypoxia inducible factor 1 (HIF1)-pathway. The hallmark *tissue invasion* is implemented to present in the sum of all matrix metalloproteinases in the model and therefore symbolizes the level of invasion and metastasis process and is directly embedded within the MAPK-, transforming growth factor beta (TGFbeta)-, insulin-like growth factor 1 receptor (IGF1R)-, and NfκB-pathway. The hallmark *evading apoptosis* is defined as the sum of anti-apoptotic factors over pro-apoptotic factors in the model to represent the potential for evading apoptosis in the model and is directly connected within the Death-Receptor-, IGF1R-, Toll-like Receptor (TLR)- and COX-pathway. The hallmark *sustained angiogenesis* is also defined as the sum of pro-angiogenesis factors over anti-angiogenesis factors to symbolize the potential for sustaining angiogenesis in the model and is directly implicated within the VEGF- and fibroblast growth factor (FGF)-pathway. All four hallmarks are defined as pseudo component in the model (Table 2). The mass action law is applied to the equations for integrating these four hallmarks into the NSAID model (see Materials and Methods). The objective of this type of modeling is to reflect the important role of COX-pathway in the cancer development and to highlight its ability to influence and attribute the tumorigenesis process in different aspects. Currently, the influence degrees of different components on hallmarks within the hallmark equations are not taken into consideration; therefore, all coefficients in the hallmark equations are set to 1 (see Materials and Methods).

**Table 2:** Hallmark Implementation.

**Hallmark**	**Implementation**	**References (PudMed)**
Evading-apoptosis (EA)	EA = (TNF1:TNFRSF1B + P-IGF1R:IGF1 + P-IGF1R:IGF2 + BCL2L1 + BCL2 + MCL1) / (1+ FAS:FASLG + TNFSF10:DR4_5 + TNF1:TNFRSF1A + BAX + BAK1 + PMAIP1 + BBC3)	14634624,17846171,21608150,20182539,8524870
Proliferation (P)	P = EIF4E + P-MYC:MAX + URGCP + MKI67 + TRIM21 + MYBL2 + TOP2A + STK6 + PLK1 + FOXM1 + PCNA	15184677,10430922,12454650,12620412,11018017,17217616
Sustained-angiogenesis (SA)	SA = (P-FGFR:FGF + P–KDR_dimer:VEGFC + P–KDR_dimer:FIGF + P–KDR_dimer:VEGFA + IL8 + GM-CSF + TIMP) / (1 + CD36:THBS1)	18560389,17933680,20010945,21248359,21742222
Tissue-invasion (TI)	TI = MMP7 + MMP9 + MMP2 + MMP1 + MMP13 + MMP10	14967450,16680569,11349215,11344033

DR4_5 indicates a component-entity in the model containing DR4 and DR5. P-IGF1R means the phosphorylated form of receptor protein IGF1R and P-IGF1R:IGF1 the complex of ligand receptor binding. P–KDR_dimer means the phosphorylated form of dimerized receptor KDR. TNF: tumor necrosis factor; TNFRSF1A (B): tumor necrosis factor receptor superfamily, member 1A (B); IGF1R: insulin-like growth factor 1 receptor; IGF2: insulin-like growth factor 2; BCL2L1: BCL2 like 1; BCL2: B-cell CLL/lymphoma 2; MCL1: myeloid cell leukemia sequence 1; FAS: Fas (TNF receptor superfamily, member 6); FASLG: Fas ligand (TNF superfamily, member 6); TNFSF10: tumor necrosis factor (ligand) superfamily; member 10; DR4_5: death receptor 4 and 5; BAX: BCL2-associated X protein; BAK1: BCL2-antagonist/killer 1; PMAIP1: phorbol-12-myristate-13-acetate-induced protein 1; BBC3: BCL2 binding component 3; EIF4E: eukaryotic translation initiation factor 4E; MAX: MYC associated factor X; PCNA: proliferating cell nuclear antigen; KDR: kinase insert domain receptor; GM-CSF: granulocyte-macrophage colony-stimulating factor; TIMP: TIMP metallopeptidase inhibitor; CD36: CD36 molecule; THBS1: thrombospondin 1; MMP: matrix metallopeptidase 3.

### Validation of Functional Indication of Cancer Hallmarks

In 2003, Denkert and colleagues performed an *in vitro* study to investigate the therapeutic inhibition effect between COX-isoform-specific siRNA and COX-2 selective drug NS-398 in the human ovarian carcinoma cell line (OVCAR-3) [28]. This was the first time to show that COX-2 selective drug NS-398 had clear inhibition effects for the OVCAR-3 cell proliferation, whereas the COX-isoform-specific siRNA did not exert any proliferation inhibition effect on this cell line. In order to test the quality of our NSAID model and the implementation of cancer hallmarks, we initialized the NSAID model with the gene expression data of the OVCAR-3 cell line (see Materials and Methods). In this way, we expected that the model should be able to reflect the dynamic behavior of the OVCAR-3 cell line during the *in silico* simulation. Afterwards, we conducted the Flux-Comparative-Analysis (FCA), which is focused on the flux comparison between a tumor state (control state) and a therapeutic intervention state (perturbation state) (see Material and Methods). In this case, we investigated the state of Tumor + COX-2 siRNA vs. Tumor state (CT comparison), and the state of Tumor + NS-398 vs. Tumor (NT comparison), in order to validate whether the NSAID model could reveal the differences of these two types of therapeutic inhibition as Denkert et al. [28] demonstrated in their study.

**Figure 2:**
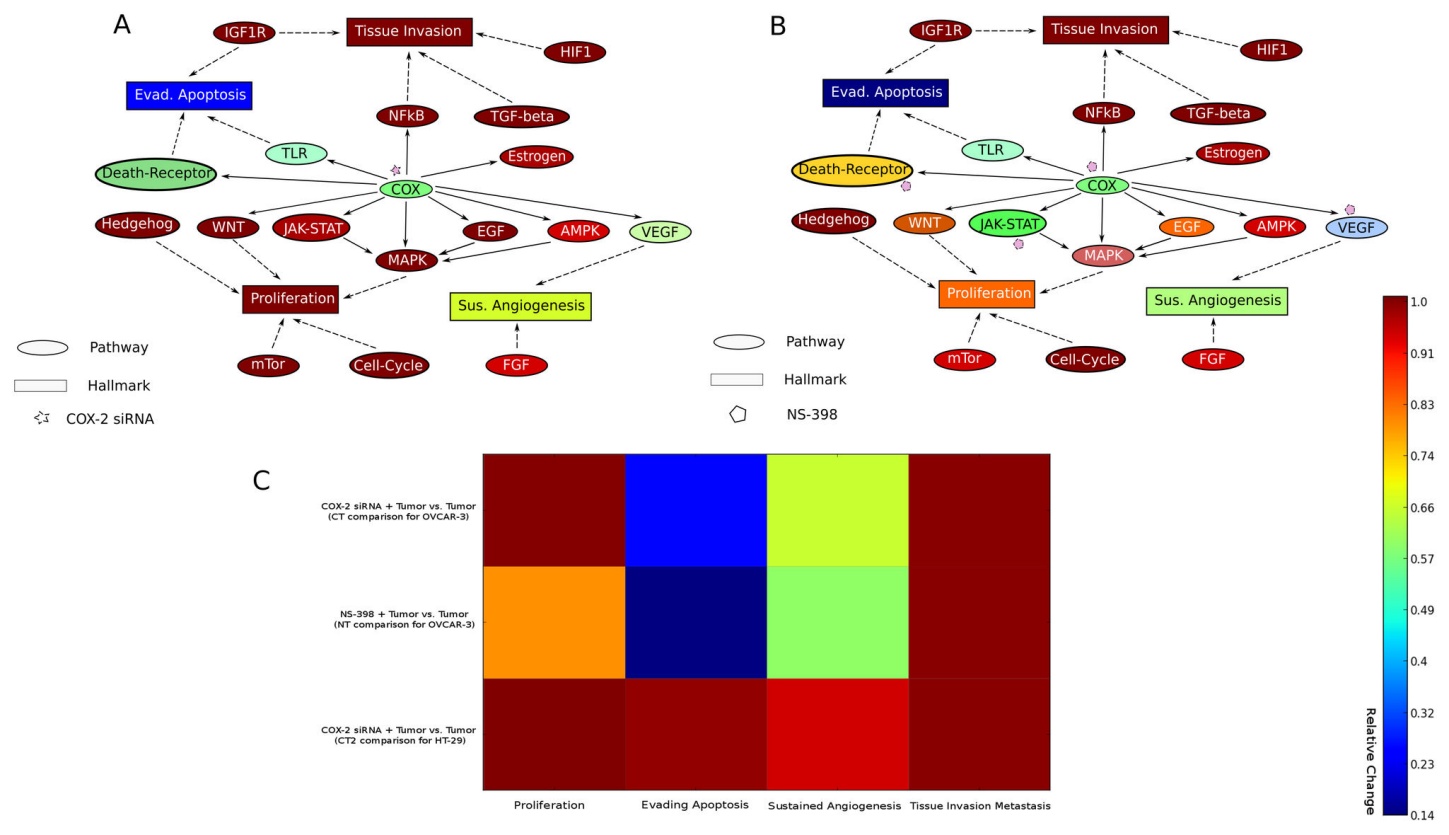
Simplified visualization of COX-2 inhibitions within the NSAID model network. **A**: Inhibition of COX-pathway via COX-2 specific siRNA interference. **B**: Inhibition of COX-pathway via the drug NS-398 (COX-2 selective inhibitor). **C**: Heatmap result of FCA includes CT comparison (first row), NT comparison (second row) and CT2 comparison (last row). The color bar is designed for **A**, B and C and color ratios of cancer hallmarks are the results of FCA analysis, which is explained in the Materials and Methods with Figure 5.

The FCA result shows that the hallmark proliferation remains the same in the CT comparison, whereas in NT comparison, the hallmark proliferation is reduced to 79.8% of the proliferation from the tumor state (Figure 2), which indicates that the NSAID model can alter the inhibition effect between COX-2 siRNA and drug NS-398 regarding the OVCAR-3 cell line. The FCA also reveals that both types of inhibitions can lead to considerable reduction of signaling path, starting from PGG_2_ to PGE_2_, which results in decreasing of signaling crosstalk of COX-pathway with many other signaling pathways. However, this type of decrease in signaling crosstalk does not exert great impact in the model, because the PGE_2_ functional dependent receptors EP1-4 are low (P<0.001) expressed in the OVCAR-3 cell line. Therefore, only targeting COX-2 in OVCAR-3 cannot reduce the high cellular activities of signaling pathways such as MAPK-, WNT- and NfκB-pathway (Figure 2A), which leads to the high expression level of several transcription factors including v-myc myelocytomatosis viral oncogene homolog (MYC), jun proto-oncogene (JUN), fbj murine osteosarcoma viral oncogene homology (FOS), and specificity protein (SP) 1,3. These essential transcription factors are known to be involved in diverse signaling pathways including MTOR-, WNT-, and Hedgehog pathway to sustain the high cellular proliferation. In contrast, the drug NS-398 simultaneously inhibits COX-2, VEGF, interleukin (IL) 1 and tumor necrosis factor (TNF) [29], which leads to reducing cellular cycle activity, signal within VEGF-, TLR- and JAK_STAT pathway and signals transduced by phospholipase C gamma (PLCG) and PTK2 protein tyrosine kinase 2 (PTK2) so that the cellular proliferation can be reduced during in silico simulation (Figure 2B) (The mathematical implementation of this drug is explained in the Materials and Methods). Furthermore, the hallmark of evading apoptosis is reduced at 14.4% and 25.8% in the CT and NT comparison respectively, because in both cases of inhibition, the FCA reveals that activities of TLR- and Death-Receptor-pathway have been reduced (Figure 2). The FCA also shows that due to the activity reductions of the VEGF- and FGF-pathway, the hallmark of sustained angiogenesis is reduced to 59.8% and 65.6% in both cases (Figure 2). These results suggest that both COX-2 siRNA and drug NS-398 can successfully reduce the COX-2 cellular function, which is involved in the pathological processes of evading apoptosis and sustained angiogenesis. However, the drug NS-398 can exert a better inhibition effect than the COX-2 siRNA does by reducing both pathological processes regarding this cancer cell line. The hallmark of tissue invasion and metastasis remains unchanged in both CT and NT comparisons.

In 2006, Strillacci and colleagues applied a RNA interference technique to knock down the overexpressed COX-2 in colon cancer cell lines (HT-29) and found that this COX-2 knockdown did not exert any effect on HT-29 cell proliferation [30]. Furthermore, at the same time, Charames and Bapat conducted siRNA experiment to inhibit the expression level of COX-2 in HT-29 cell lines and revealed that this type of siRNA inhibition did not have effect on HT-29 apoptosis [31]. In order to further validate the hallmarks in the NSAID model, we initialized the NSAID model with the gene expression data of the HT-29 cell line (see Materials and Methods) and performed FCA analysis for the HT-29 cell line of the state of Tumor + COX-2siRNA state vs. Tumor state (CT2 comparison). The result shows that the hallmarks of proliferation remained unchanged, because the low expression of PGE_2_ cognate receptor EP1-4 cause the less impact effect of dramatical signal reduction of COX-pathway in the model, many important transcription factors including SP3, c-myb viral oncogene homolog (MYB), GLI family zinc finger (Gli) and catenin (cadherin-associated protein), beta (CTNNB) involved in MAPK-, Hedgehog- and WNT pathway are not affected by this COX-2 siRNA interference in HT-29 cell line. Moreover, evading apoptosis is only slightly reduced (99.3% of the tumor state) (Figure 2C), because many caspase (CASP) proteins in the model maintain a high cellular activity. These results are qualitatively in agreement with results of both studies of Strillacci et al. [30] and Charames and Bapat [31]. Based on the fact that COX-2 siRNA interference cannot affect the cellular proliferation in OVCAR-3 and HT-29 cancer cell lines, we would like to suggest the restriction of COX-2 siRNA interference for cell lines and xenografts that have a low expression level of EP1-4 receptors. However, this suggestion should be further verified in future studies.

### Analysis of COX Based Synthetic Lethality for Breast, Colon and Lung Tumor

Synthetic lethality describes the relationship of a gene pair, when the simultaneous mutations of both genes can lead to cell death, while the mutation of each gene is still compatible to the cell viability [32]. This concept of synthetic lethality could provide essential molecular information for developing high quality of anti-cancer drugs, which can enhance the on-target efficiency and reduce the off-target effects to the minimum. Based on the anti-tumor effect of the COX-pathway, many preclinical studies have indicated that the treatment by inhibiting COX-2 (key component of COX-pathway) and a receptor tyrosine kinase such as EGFR, v-erb-b2 erythroblastic leukemia viral oncogene homolog 2 (ERBB2), could yield additive effect, which is far more effective than either single agent alone [33–35]. Therefore, we aimed to apply the NSAID model to investigate impact of the COX-based synthetic lethality with all receptor tyrosine kinases defined in this model for different tumor types. We utilized the gene-expression data of 60 tumor cell lines that are provided by the cancer genome atlas (https://tcga-data.nci.nih.gov) and performed *in silico* simulations to investigate the COX-2 based combination inhibition. These tumor cell lines include 20 breast tumor cell lines, 20 colon tumor cell lines and 20 lung tumor cell lines (Supplementary Information S3). All combination-inhibitions of COX-2 and receptor tyrosine kinases within the NSAID model were analyzed for each tumor cell line. The analysis method is based on the FCA. In this case, the control state is the steady state which the NSAID model reaches during *in silico* simulation with the gene-expression data initialization of corresponding tumor cell lines. The perturbation state is the steady state which the NSAID model reaches with the same data initialization and additional combined inhibition of COX-2 and a receptor tyrosine kinase. The readout components of FCA are the integrated four cancer hallmarks in the model. Each combination-inhibition (such as COX-2+EGFR and COX-2+ERBB2) presents a type of therapeutic perturbation for a tumor cell line.

**Figure 3:**
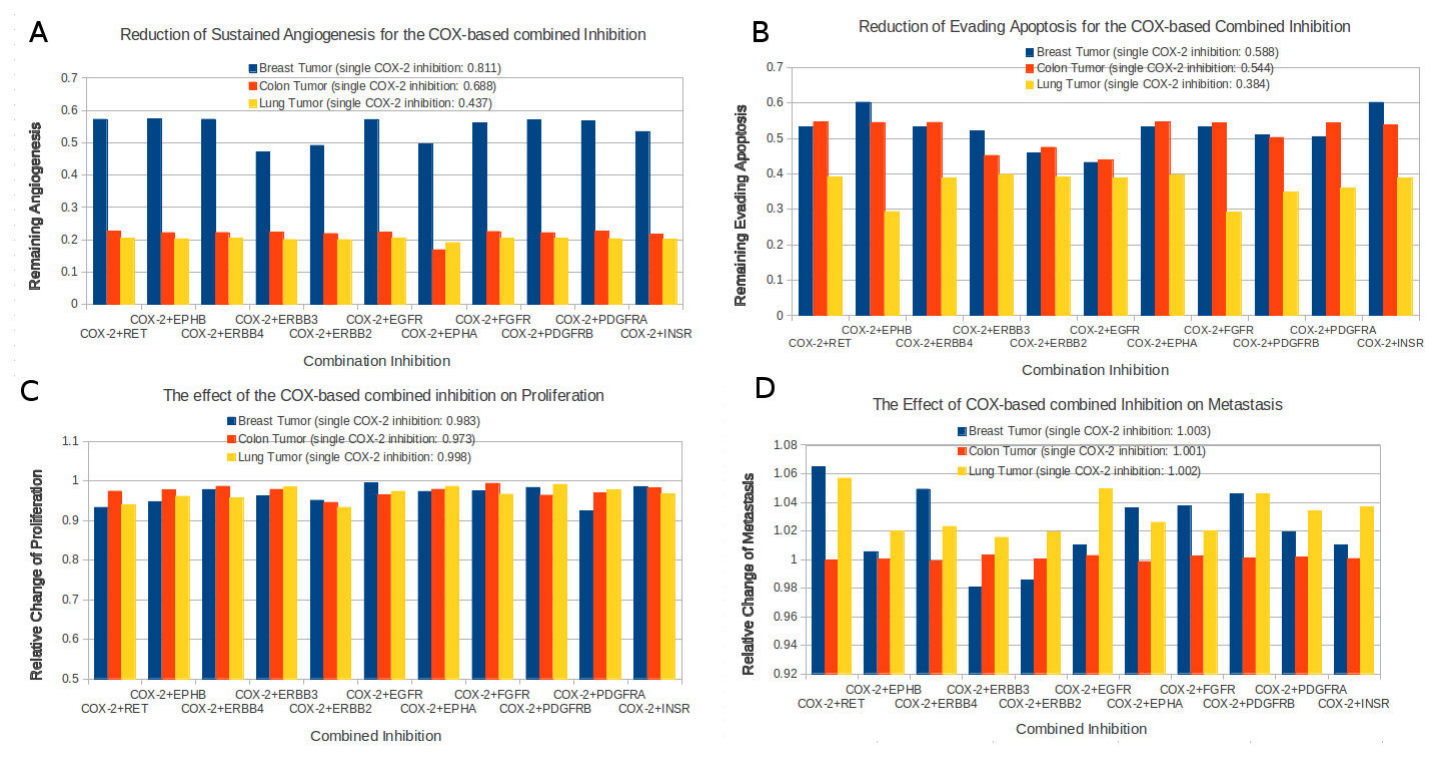
Effect of the COX-based combined inhibition on cancer hallmarks. Angiogenesis (**A**), Evading Apoptosis (**B**), Proliferation (**C**) and Metastasis (**D**). The blue, red, and yellow columns in each histogram indicates averages of the relative changes of cancer hallmarks for 20 breast carcinoma samples, 20 colonrectal carcinoma samples, and 20 lung carcinoma samples respectively. The relative change of each cancer hallmark refers the comparison between therapeutic perturbation state (COX-based inhibition) and control state (tumor state), which is calculated by the Flux Comparative Analysis (FCA).

Angiogenesis is the process of new blood vessel formation and in the course of solid tumor development, tumor tissue such as breast, colon and lung becomes highly dependent on angiogenesis for maintenance and progression [36]. The combination inhibitions of COX-2 and receptor tyrosine kinases could achieve more significant reductions of angiogenesis than single COX-2 inhibition during *in silico* simulation, especially for colon and lung tumor cell lines (Figure 3A), where combination inhibitions of COX-2 lead to less than 30% remaining angiogenesis processes (68.84% and 43.72% remaining angiogenesis for single COX-2 inhibition in colon and lung tumors respectively). These results indicate that the combination inhibition of COX-pathway and a receptor tyrosine kinase can dramatically reduce the angiogenesis, which leads to growth inhibition of colon and lung tumor. These results act in concert with the study of Tuccillo et al. [37], who examined the additive effect of the anti-cancer drug-combination of ZD6474 (a EGFR and VEGFR inhibitor) and SC-236 (a selective COX-2 inhibitor) on the xenograft models of lung and colon cancer, and these results are also in agreement with the study of Mann et al. [33] and Tortora et al. [34]. Both studies applied the combined treatment for inhibiting COX-2 and ERBB2, and COX-2 and EGFR to exert the anti-angiogeneic effect on colon cancer cell lines and xenograft models.

For breast tumor, the combination inhibitions of COX-2 and receptor tyrosine kinases could reach between 40% and 60% remaining angiogenesis, which are far better than 81.13% remaining angiogenesis of a single COX-2 inhibition (Figure 3A). These additive effect suggests that the combined treatments of COX-2 and receptor tyrosine kinases might be a effective therapeutic strategy for breast tumor treatment. However, compared to other two tumors, the breast tumor shows stronger resistance to COX-2 based combination inhibitions (Figure 3A). We compared the signal intensity among pathways in the NSAID model to investigate which pathways might possess high activity to contribute this type of inhibition resistance. We found out that under the combination inhibition, in average, breast tumor cell lines still possess relatively higher activities (> 1.4 fold) of Hedgehog-pathway (1.75 fold), EGFR-pathway (1.64 fold), JAK/STAT-pathway (1.49 fold), WNT-pathway (1.43 fold), compared to colon tumor cell lines; while compared to lung tumor cell lines, breast tumor cell lines possess relatively higher activities (> 1.4 fold) of JAK/STAT-pathway (1.67 fold), cell-cycle-pathway (1.59 fold), ERBB-pathway (1.5 fold).

Apoptosis, the process of programmed cell death, is a critical cellular mechanism for enabling the efficient removal of superfluous, damaged or infected cells. The disturbances to the apoptotic machinery can lead to excessive cell survival or cell death, which can result a number of pathological conditions including tumorigenesis [38,39]. All combination inhibitions of COX-2 and receptor tyrosine kinases can reach below 61% remaining evading apoptosis for these three tumor types. Especially for the lung cancer, the remaining evading apoptosis is even below 40% (Figure 3B). Interestingly, in comparison with average remaining evading apoptosis by single COX-2 inhibition of these three tumor types, the combination inhibitions of COX-2+ERBB2, COX-2+ERBB3, COX-2+EGFR and COX-2+PDGFRB (platelet-derived growth factor receptor beta), show clear additive effect for breast and colon tumors, whereas for lung tumor, the combination inhibition of COX-2+EPHB (EPH receptor) and COX-2+FGFR have a clear additive effect. All other combination inhibitions for these three tumor types remain almost the same as the single COX-2 inhibition, which indicates no additive therapeutic effect of those combination inhibitions. Interestingly, the combination inhibition of COX-2+INSR shows even higher evading apoptosis than the single COX-2 inhibition. The reason for this is that the inhibition of INSR strongly reduces the insulin signaling which leads to decrease of phosphorylated protein kinase C (PKC). The decrease of active PKC results in a signal reduction of NfκB-pathway, whose downstream target genes include different pro-apoptotic ligands and receptors such as Fas (TNF receptor superfamily, member 6) (FAS), TNF-1, tumor necrosis factor receptor superfamily, member 1A (TNFRSF1A).

Proliferation is the cellular process for cell growth and development. With the sufficient nutrition and suitable micro-environment, the rate of cellular proliferation becomes stable, which leads to an increase of cell population under the control of the normal cellular system. The primary task of cancer development is to stay with the pathological state of highly controlled proliferation. Unfortunately, for these three types of tumors, all combination inhibitions of COX-2 and receptor tyrosine kinases can only exert very little effect on the proliferation process, which is implied by the result that remaining proliferation process is higher than 90% for all those combination inhibitions. Furthermore, none combination inhibition does not perform clearly better (reduction 30%; p<0.05) proliferative inhibition than the single COX-2 inhibition of corresponding tumor type during *in silico* simulation (Figure 3C). Only three combination inhibitions showed little improvement (reduction 4%; p<0.05) in comparison with a single COX-2 inhibition. They are COX-2+RET (ret proto-oncogene), COX-2+ERBB2 and COX-2+PDGFRA.

Tissue invasion, also called metastasis, is the primary course of cancer mortality and is also one of the most pertinent hallmarks of cancer from a therapeutic perspective [40]. During the *in silico* simulation, all combination inhibtions except three combination inhibitions (COX-2+ERBB2 for breast tumor, COX-2+ERBB3 for breast tumor and COX-2+EPHA for colon tumor), do not have any effect on the metastasis. Nor do the single COX-2 inhibitions have any effect on the metastasis for the three tumors in the model. The combination inhibitions COX-2+ERBB2 and COX-2+ERBB3 can exceptionally exert little effect on the metastasis for breast tumor, which are reflected by the ~ 98% remaining metastasis after inhibition. And the combination inhibition COX-2+EPHA can have even little inhibition effect with reaching ~ 99% remaining metastasis.

### MiRNA Regulation on COX Pathway and The Potential for Biomarker Discovery

**Figure 4:**
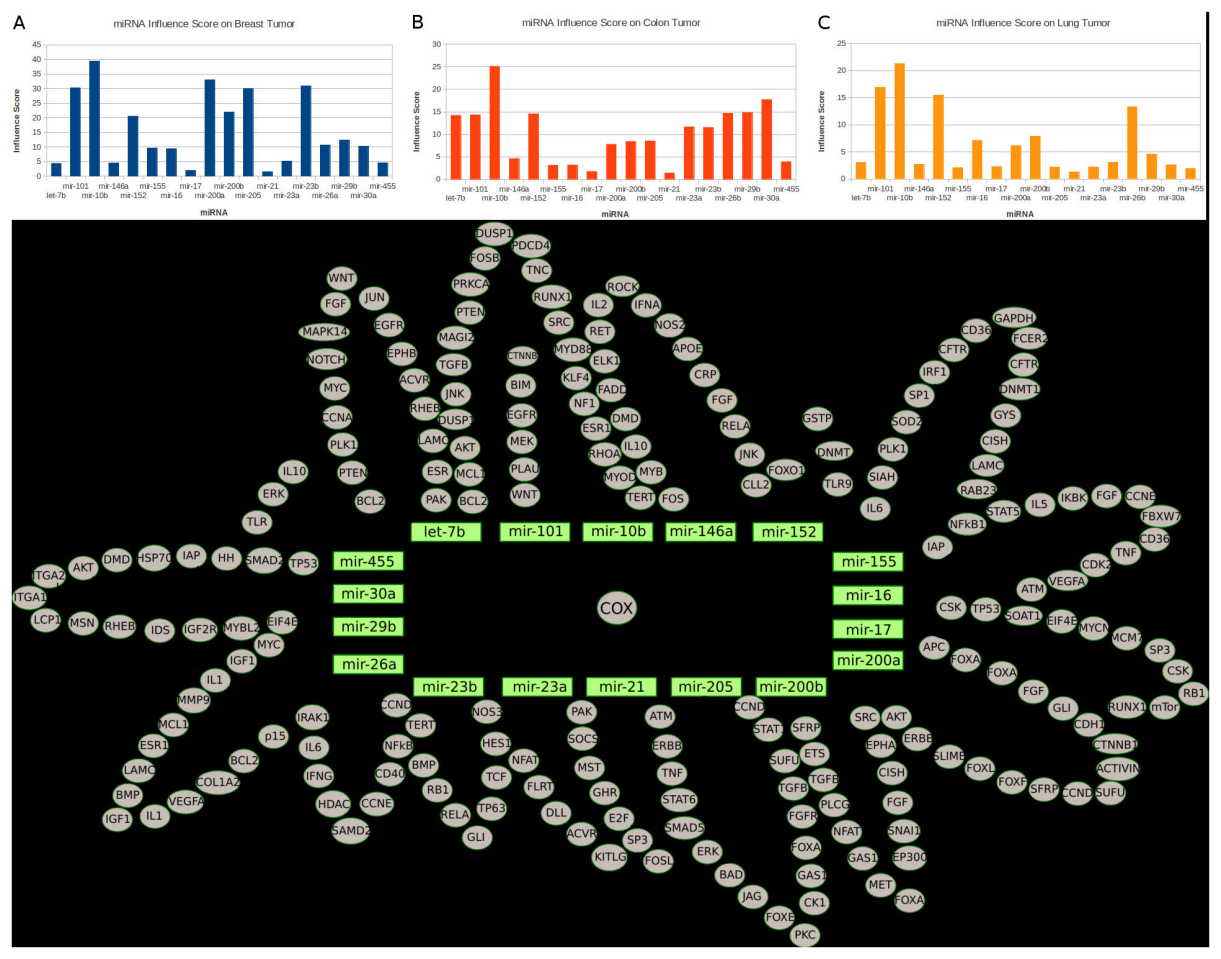
Influence-score analysis of the 18 miRNAs on breast- (A), colon- (B), and lung- (C) tumor; D. The visualization of miRNA regulation network.

In recent years, many studies revealed that miRNAs play important roles in diverse cellular processes such as angiogenesis, proliferation, cell cycle and apoptosis, therefore contribute to the tumorigenesis [41,42]. Some recent studies provide evidence that the lower expression level of certain miRNAs (miR-101a and miR-199a) is associated with high COX-2 expression during different cellular processes [43,44]. We incorporated 18 miRNAs with their relevant validated target information (Supplementary Information S4) into the NSAID model, whose targets all include the key component COX-2. The miRNA modeling approach is introduced in the previous study [45]. We utilized the published miRNA expression data for the same three tumor types from cancer genome atlas and investigated if the overexpression of those miRNAs could yield satisfactory therapeutic effect with the in silico approach (FCA). For each miRNA, we created a control state (steady state of the NSAID model with gene expression data initialization) and perturbation state (steady state of the NSAID model with the same initialization and additional 100-fold higher miRNA expression), and we repeated this FCA of miRNA overexpression for the same 60 tumor cell lines. The objective of this FCA is to investigate the system-level influence of each miRNA when its overexpression represses the commonly overexpressed COX-2 and additionally reduces the expression level of other targets to yield the effect of “One Hit Multiple Targets” [46]. Based on FCA results, we performed the Wilcoxon signed-rank test for calculating the influence score of each miRNA for 60 tumor cell lines (see Materials and Methods), the score shows how the overexpression of miRNA can exert impact on the model. Afterwards we averaged scores of miRNAs according to the tumor type (Figure 4. A, B and C).

It is noteworthy that many tumor suppressor miRNAs such as mir-101, mir-10b, mir-200a, mir-205, mir-23 in breast tumor have high influence scores (>20) (Figure 4A). For example, mir-10b is the major contributor for the breast cancer metastasis [47]; mir-101 is the key regulator of autophagy and modulates the cancer epigenome [48,49]; mir-205 is responsible for suppression of cell growth and invasion in breast tumor [50]. The FCA result reveals that the overexpressions of those miRNAs have a common inhibition effect on COX-pathway, which leads to high impact on the NSAID model. This result is in agreement with the fact of the important role which this pathway plays during breast cancer development [51,52].

Interestingly, we found out that many miRNAs that are typically upregulated in colon cancer stem cells have low influence scores (<4) (Figure 4B), such as mir-155 [53], mir-16 [53], mir-17 [54] and mir-21 [55] (Figure 4B). This result implies that the overexpression effect of these typically uprelated miRNA in colon cancer cellular system might be saturated, therefore the further overpression of them could only exert little impact on the NSAID model. Moreover, this result might also imply a less significant role of the COX-pathway in colon cancer stem cells. In lung tumors, only 4 miRNAs (mir-10b, mir-101, mir-152 and mir-26b) have relatively high influence scores (13<score<23) (Figure 4C). This result indicates the tumor suppressor roles of those miRNAs in lung tumor, which act in concert with the results provided by different independent studies conducted by Zhang et al. [56] and Arora et al. [57]. The rest of miRNAs with low influence scores (<7) indicate the saturation of an overexpression effect in the NSAID model as stated similarly for colon cancers. In addition, we found that some miRNAs such as mir-10b and mir-21 have extremely high (>20 score; p=0.0034) and low (<3 score; p=0.0021) influence scores in all these 60 tumor cell lines respectively. Because of their putative role as biomarker validated in different independent studies [58–60], we would like to draw the conclusion that those miRNAs with extremely high or low influence scores should be considered as miRNA biomarker of individual tumor cell lines.

## Materials and Methods

### Implementation of Cancer Hallmarks

The four cancer hallmarks (sustained angiogenesis, tissue invasion and metastasis, proliferation and evading apoptosis) are defined as pseudo components in the NSAID model and they are also considered as readout component for the Flux Comparative analysis (explained below). Each of them should be able to summarize the signal from signaling pathways and presents a developmental aspect of the cancer cell physiology. The modeling implementation of these four cancer hallmarks is listed below with the literature references and the mass action law is applied for the integration implementation. Currently, the influence degrees of components on cancer hallmarks have not been taken into consideration.

### Flux Comparative Analysis (FCA)

The flux of a biochemical reaction defines the mass-flow from substrates into products. For example, a substrate A is converted into a product B in a reaction R. For simplicity, let us denote c[A] and c[B] concentrations of A and B respectively. The initial concentration of c[A] = m and c[B] = 0. By applying the mass action law with a kinetic parameter k, after the reaction R occurs once, the c[B] = m * k and c[A] = (1-m) * k. Now, the c[B] can describe the quantity of the flux of the reaction R. In this case, the product B is a readout component for the implemented model. In this way, we assign all reactions in the model with the standard rate law, mass-action kinetics, in order to take the pragmatic solution for model simulation (Table 3).

**Table 3:** Summary of mathematical implementations of reactions in NSAID model.

**Reaction**	**Biochemical presentation**	**Kinetic rate law**	**Parameter Value**
Transcription	G → m	v = [G] * K_transcription_	0.25
Translation	m → P	v = [m] * K_translation_	0.35
Decay	S1→	v = [S] * K_decay_	0.1
Complex Formation	S1 + S2→ S1:S2	v = [S1]* [S2]* K_complex_	0.7
Translocation	S1(A)→ S1(B)	v = [S1(A)]* K_in_ – [S1(B)]*K_out_	0.5
Phosphorylation	P→ phos-P (with Enzyme a. Inhibitor)	v = [P] * [E] * K_e_/(1+K_i_) * [I]	0.35/0.2
Activation	P→ active-P (with Enzyme a. Inhibitor)	v = [P] * [E] * K_e_/(1+K_i_) * [I]	0.4/0.2
miRNA regulation	m→ (miRNA regulation)	v = [m] * [miRNA] * K_mi_	0.01

G: Gene; m: mRNA; P: Protein; S: Substance. A and B stand for different location.

**Figure 5:**
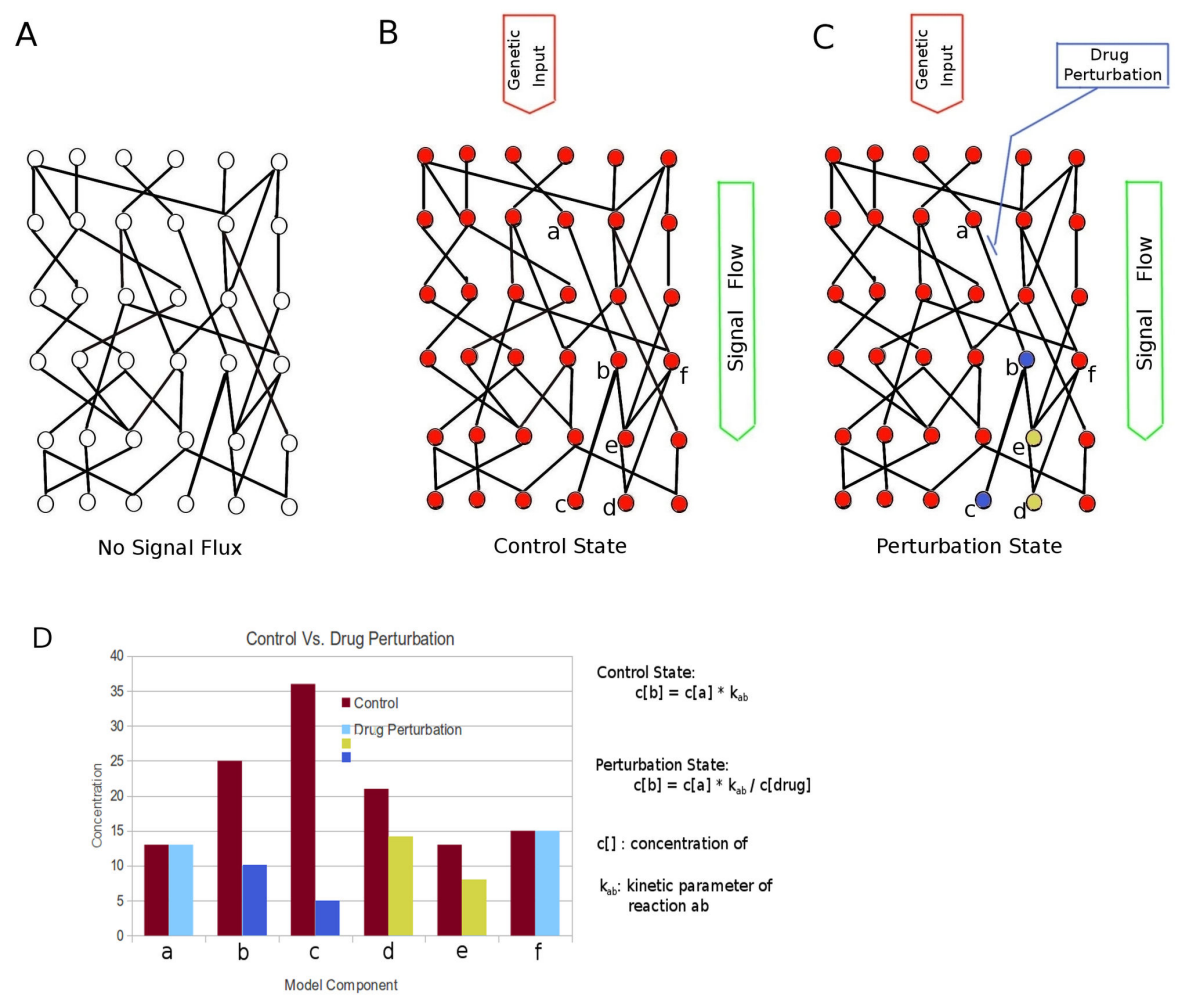
Visualization of the Flux-Comparative-Analysis. **A**: empty flux state of model network; **B**: control state of model network; **C**: perturbation state (drug inhibition) of model network. **D**: the comparison of model components between control state and perturbation state. The mathematical implementation of both states in the model is described.

As the name of this approach says, during simulation, we compare the flux of the readout component in the implemented model from a control state with the flux of the same component from a perturbation state. We use the four cancer hallmarks in the model as readout components to investigate if any kind of therapeutic perturbation could influence these readout components. The goal of this approach is to relatively reveal the effect of the perturbation state (e.g. drug treatment condition) versus the control state (e.g. pathological cellular condition) on the model, in order to predict the therapeutic effect of different kinds of perturbation. Figure 5A is a model network with empty signal flux. The Figure 5B and 5C symbolize the control and perturbation states of the same model network with the same flux input based on the genetic information (currently the gene expression data). After the flux within the model system reaching steady state, the corresponding components are analyzed (Figure 5D, histogram), which shows whether or how this kind of perturbation could influence the key readout component of the model. As shown in the Figure 5D, the component c in the control state has the concentration of 36 (a.u.) and in the perturbation it has the concentration of 5 (a.u.), so the FCA result of component c is 0.14. Similarlz, FCA results of components a, b, d, e and f are 1.0, 0.40, 0.67, 0.62 and 1.0, respectively (Figure 5D). Therefore, the absolute value of genetic information is irrelevant for the FCA analysis, only the data proportionality is essential for this approach. The simulation procedure is based on the Petri net extension described in the previous study [45].

### Mathematical Implementation of COX-isoform Specific siRNA and NS-398 Drug Effect

The drug effect of COX-2 specific siRNA is concentration reduction of COX-2 mRNA, while the drug effect of the drug NS-398 consists of effects generated from four inhibitors that promote the protein degradation processes of COX-2, VEGFA, IL1 and TNF respectively. This type of drug effect implementation is inspired by the *in vitro* study of Hidvegi et al. [61], where the authors successfully demonstrated in a mouse model that the drug carbamazepine can reduce the alpha-antitrypsin Z (ATZ) by promoting its degradation, which leads to reduction of hepatic fibrosis. Both mathematical implementations are listed in Table 4.

**Table 4:** The difference of mathematical implementation of different states for FCA analysis.

**CT Comparison**	**Tumor + COX-2 siRNA State**	**Tumor State**
Kinetic Rate Law	v=[COX-2(mRNA)] * K_translation_/[siRNA]	v = [COX-2(mRNA)] * K_translation_
**NT Comparison**	**Tumor + NS-398 State**	**Tumor State**
Kinetic Rate Law	v = [COX-2(Protein)]* K_decay_* [Inhibit1]	v = [COX-2(Protein)]* K_decay_
	v = [VEGF(Protein)]* K_decay_* [Inhibit2]	v = [VEGF(Protein)]* K_decay_
	v = [IL1(Protein)]* K_decay_* [Inhibit3]	v = [IL1(Protein)]* K_decay_
	v = [TNF(Protein)]* K_decay_* [Inhibit4]	v = [TNF(Protein)]* K_decay_

The effect of drug NS-398 is now composed of the effect of inhibit1-4.

### Model Initialization with Gene-Expression Data

The model contains different object types including gene, RNA (mRNA, miRNA), protein, complex compound and pseudo-object and each type is associated with a corresponding Id. For instance, gene- and mRNA-object are ensembl-id; protein- and compound-object are associated with uniProt-id and ChEBI-id respectively; miRNA- and miRNA gene-object are associated with the miRNA accession; other objects are associated with internal model id. All objects except the gene object are set to 0. During the simulation process, the model signal is only generated from the transcriptional level and forwarded to the translational level. Afterwards, the signal can be propagated to the rest of model.

The initialization procedure is preformed according to ensembl-id of gene objects in the model; all gene objects in the model are iteratively assigned with a corresponding gene expression value according to this Id. Since the value of gene expression data from Cancer Genome Atlas is log-ratio, we have to modify the data by exponentiation to retrieve originally measured gene expression values, because negative log-ratio is not suitable for Petri net simulation. Afterwards the aforementioned initialization procedure (pseudo-source code) is applied to initialize the NSAID model.

Pseudo-source Code:

1. m ← signaling model

2. Initialize(m){

3. if gene expression data is available{

4. gene-entities ← m.getAllGene_Entities()

5. for gene-entity in gene-entities{

6. set gene-entity with data according to the Ensembl id

7. }

8. }

9. if miRNA expression data is available{

10. miRNA-entities ← m.getAllmiRNA_Entities()

11. for miRNA-entity in miRNA-entities{

12. set miRNA-entities with data according to the miRNA id

13. }

14. }

15. set all other entities in the model to zero.

16. }

17. simulate(m){ # signal flux propagation process on the Petri net extension [45]

18. }

### Calculation of the miRNA Influence Score

Suppose the model having n components and C_x_ (1 < =x<=n) is defined as the concentration of one model component. Let us denote the arrays C = [C1, C_2_, C_3_ … C_n_] and C' = [C'_1_, C'_2_, C'_3_ … C'_n_] are concentrations of all model components in the control state and perturbation state respectively, with regards to the FCA analysis. The p-value (P) calculated by the Wilcoxon signed-rank test(C, C') implies the system-level impact between model components in both states due to the miRNA regulation. (It is noteworthy that p-value calculated in this way should not be considered a possibility of obtaining a statistical test for acceptance or rejection of null hypothesis.) 

miRNA influence score (MIS) = (-1) * log(P, 10)

### Model Availability

The NSAID model is available under the ftp://138.245.80.137/NSAID_model.xml in the form of XML.

## Discussion and Conclusion

The general aim of this study is to assess the feasibility as to whether the molecular-based model construction can be applied for the purpose of therapeutic development including new potential targets identification and high quality biomarker discovery. Thus, in this study the first molecular NSAID model was introduced, whose construction is based on literature references regarding the COX-pathway and its related pathways. This model integrates four cancer hallmarks to realize a biological organization principle for tumorigenesis, which is a multiple-step process in human cells [22,23]. Each cancer hallmark in the NSAID model should reflect a corresponding developmental aspect of cellular malignant transformation. This study has employed the data from different in-vitro studies to validate functional indications of these cancer hallmarks and reached concert with the results of those in-vitro studies [28,30,31]. In addition, we propose a criterion for application-restriction of COX-isoform specific siRNA interference in tumor cell lines. The result also indicates that the NSAID model could inherit the dynamic behavior of corresponding tumor cellular systems and react with a similar response when facing the therapeutic intervention (siRNA interference and NS-398 drug), which might serve as “Virtual Patient” for prediction of therapeutic responses from an individual tumor cell line.

By applying the NSAID model, we tried to explore the novel concept of synthetic lethality related to the key component (COX-2) of COX-pathway for 60 cell lines of breast-, colon- and lung tumor. Many in-vitro and in-vivo studies provided evidence that the combined treatment by inhibiting the key component of COX-pathway and a relevant receptor tyrosine kinase such as EGFR and ERBB2, could yield significant additive therapeutic effect. Our *in silico* approach reveals that this type of combined inhibition (COX-2 and a receptor tyrosine kinase) could reach much better angiogenesis reduction than a single COX-2 inhibition for 40 cell lines of both colon- and lung-tumors, which reaches an agreement with different independent in-vitro studies [33–35,37]. In addition, we pointed out that the additive effect of combined inhibition on breast tumor should be validated by follow-up studies.

Furthermore, we integrated 18 miRNAs into the NSAID model in order to investigate miRNA regulation impact on the model system. Through the influence-score analysis of miRNA, we drew the conclusion that miRNAs with a higher influence scores have higher possibility to be tumor suppressor miRNA in the corresponding tumor, while miRNAs with lower influence scores have a higher possibility to be oncogenic miRNAs. Those miRNAs with extremely high or low influence scores are proposed to be considered a miRNA biomarker. This result of *in silico* biomarker discovery is in line with recent independent studies [58–60]. This influence-score analysis might shed light on the development of an *in silico* approach for biomarker identification at individual level. Many recent studies provide evidence about the deregulated expression profiling of miRNA in diverse cancers and elucidate that deregulation of multiple miRNAs belongs to the common scenario in cancers [41,62,63]. Therefore, the biomarker of a group miRNAs with similar functionalities should be more meaningful and significant than the biomarker of a single miRNA. Based on this fact, it is possible to extend this current *in silico* approach to identify group-wise miRNAs with extreme scores to define the group-wise miRNA biomarker. However, the exact procedure is still under investigation.

Many studies introduced mathematical models with the application of Flux-Balanced analysis and Elementary-Flux Modes [64–66]. These types of applications do not take into consideration the fact of dynamic properties which describe the physiological, developmental and pathological processes for a cellular system. In contrast, this study introduces the Flux-Comparative-Analysis (FCA) to combine the genetic input (e.g. Gene-expression data), network structure (NSAID model) and kinetic parameters of biochemical reactions to order to reflect the core aspects of cellular malignancy development and predict the drug effect and clinical outcome. The satisfactory results imply that based on mass action law the NSAID model could reach the certain approximation in order to represent the dynamics of an individual cellular system with sufficient accuracy. However, the currently applied kinetic parameters are based on the empirical experience and takes into consideration kinetic parameter information listed by the study of Papin et al. [67]. Future studies should put emphasis on detecting and measuring kinetic parameter values under different environmental conditions in order to define specific interval value with regard to different types of biochemical reactions.

Although the construction of the current NSAID model is based on literature information, there are still many limitations on it, for instance, in the model the concentrations of many metabolites including ADP, H_2_O, Orthophosphate and others, are fixed in order to prevent the signal drop-down within signaling pathways when those metabolic byproducts are running out. For all defined phosphorylation reactions in the model, we do not consider the functional difference between different phosphorylated sites within the same proteins. Furthermore, the biological functions among different paralogs such as ERK1 and ERK2, have not been considered either. Different studies show that epigenetics plays an important role in cancer biology [68,69], for instance, DNA hypermethylation and hypomethylation can be correlated with diagnosis and prognosis of cancer treatment [70–72]. However, to the present, it is still not clear how to efficiently translate those specific epigenetic information into a systemsbiological model. Currently, the different influence degrees of model components that act directly on the cancer hallmarks have not been considered. Future studies should put emphasis on this point to improve the cancer hallmark integration by recruiting cancer patients to further investigate the major NSAID drug effect and side effect with the application of this model.

Finally, the tumour-xenograft model represents the current standard for preclinical testing of anticancer agents; however, this type of model has too many limitations to remain an acceptable gateway to clinical trials [73]. This study gives a systemsbiological application-example indicating that a molecular based model containing biological information related to gene expression, gene regulation, protein interaction, signal transduction and other cellular processes, can lead to prediction of systems-level behavior of cellular system underlying an individual cell line (or patient). Despite mentioned limitations, the NSAID model with FCA might present an alternative for preclinical testing of anticancer agents related to COX-pathway to reduce expenditure of time, expenses and technical challenges.

## Supporting Information

Information S1 **A Modeling Example for the NSAID Model.** This supplementary information elucidates basic modeling principle of the connection for three layers (gene, RNA, protein) within NSAID model.
(DOC)

Information S2 **Pathway Information containing the pathway description, mechanism, transcriptional target and literature reference from all pathways defined in the NSAID model.**
(XLS)

Information S3 **The IDs of three tumor cell lines; the applied 60 tumor cell lines from three tumor types (breast, colon, and lung) are provided from cancer research organization of The Cancer Genome Atlas and the data can be downloaded from its link (https://tcga-data.nci.nih.gov)**.
(TXT)

Information S4 **The validated miRNAs’ targets that are incorporated into the NSAID model; for each miRNA’s target, there are corresponding literatures (**PubMed ID**) to support its target validation.** This table contains three columns. The first column is the name and ID of miRNAs defined in the NSAID model. The second column contains the ensembl-ID of corresponding target genes and the third column displays PubMed ID related to corresponding targets.
(CSV)
