# Supplementary material for: Modeling of Non-Steroidal Anti-Inflammatory Drug Effect within Signaling Pathways and miRNA-Regulation Pathways
Source: PLoS One. 2013 Aug 14;8(8):e72477. doi: 10.1371/journal.pone.0072477 (PMC3743815; doi:10.1371/journal.pone.0072477)
Supplement: Information S1 — A Modeling Example for the NSAID Model. This supplementary information elucidates basic modeling principle of the connection for three layers (gene, RNA, protein) within NSAID model. (DOC) [file pone.0072477.s001.doc]

Supplementary Information 1: A Modeling Example for the NSAID Model

This supplementary information elucidates basic modeling principle of the connection for three layers (gene, RNA, protein) within NSAID model.


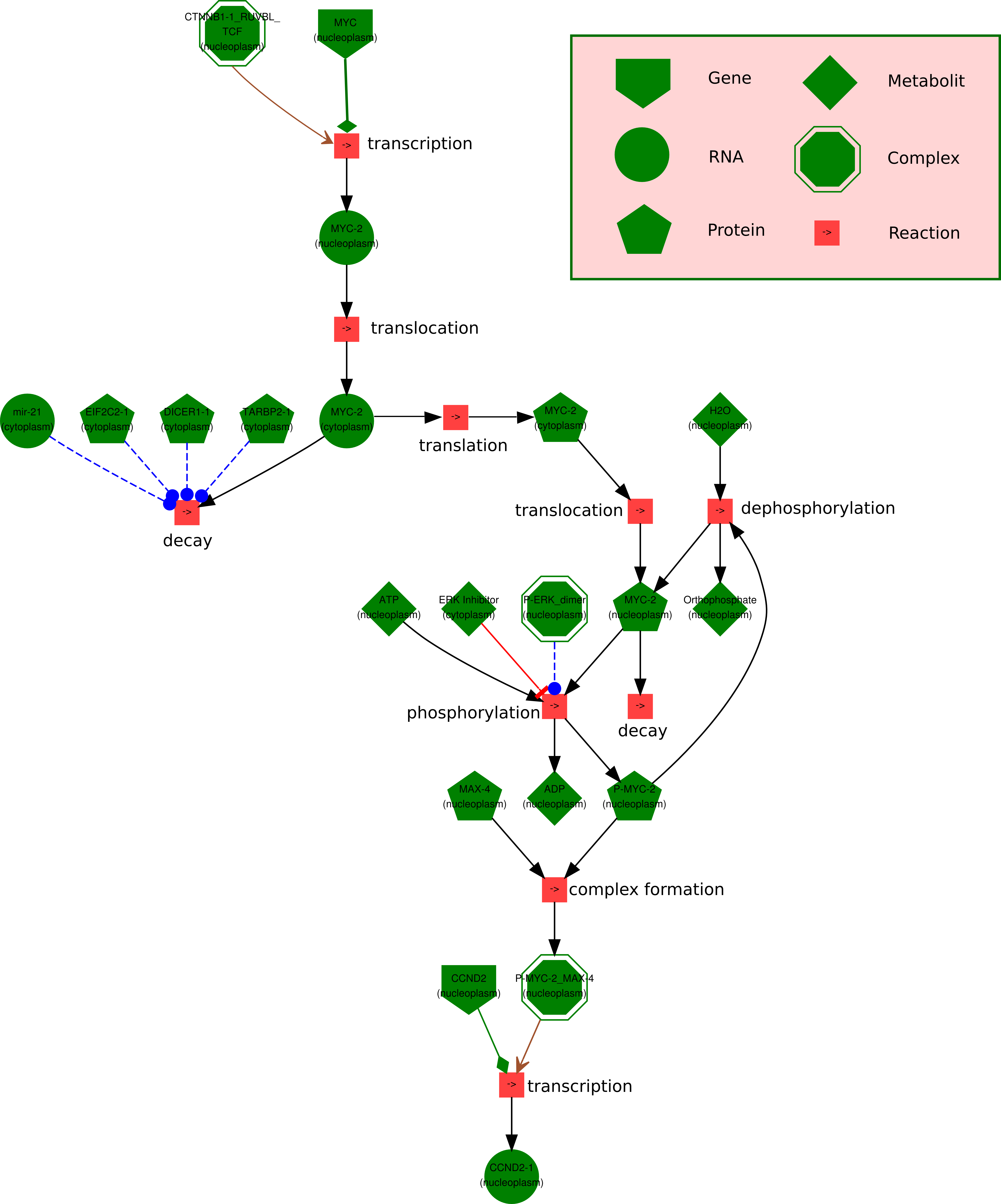
We exemplify some model components and reactions involving MYC-gene, MYC-RNA, MYC-protein and MYC-complex (Figure). At first, the MYC gene is defined in the model as a gene-object, which takes part in the transcription reaction to produce the MYC mRNA (*nucleoplasm*) under the influence of transcription factor such as CTNNB1-1:RUVBL:TCF complex. Afterwards, the MYC mRNA located in nucleoplasm is translocated into the cytoplasm, where this mRNA is under the post-transcriptional regulation of different miRNAs such as miR-21. The MYC mRNA also takes part in the translation reaction to produce the MYC protein (*cytoplasm*). This protein is later translocated into the nucleoplasm due to its famous role of transcription factor. In nucleoplasm, the MYC protein can be phosphorylated by the active ERK protein. Afterwards, the phosphorylated MYC dimerizes with its partner MAX to exert the transcriptional activity to transactivate many target genes including CCND2. Figure 1 visualizes the modeling diagram and table 1 lists mathematical implementation of defined reactions. All genes, mRNAs and proteins in NSAID model are inter-connected with each other in a similar way.

Mathematical Implementation of the modeling is based on mass action law.
